# Case Report: Autoimmune hemolytic anemia associated with ovarian teratoma in a 13-year-old: a rare paraneoplastic presentation

**DOI:** 10.3389/fped.2025.1700443

**Published:** 2025-10-27

**Authors:** Salman Althobaiti, Ahmad Assinnari, Mayes Alharbi, Razan Kurdi

**Affiliations:** ^1^ Family and Community Medicine and Medical Education Department, Faculty of Medicine, Taibah University, Medina, Saudi Arabia; ^2^ Department of Basic Medical Science, College of Medicine, Taibah University, Medina, Saudi Arabia; ^3^ Faculty of Medicine, Taibah University, Medina, Saudi Arabia

**Keywords:** autoimmune hemolytic anemia, warm AIHA, ovarian teratoma, paraneoplastic syndrome, pediatric hematology, case report, mature cystic teratoma

## Abstract

**Background:**

Autoimmune hemolytic anemia (AIHA) is a rare and potentially life-threatening condition in the pediatric population. While often associated with autoimmune or lymphoproliferative disorders, its occurrence in conjunction with benign ovarian tumors, such as mature cystic teratomas, is exceptionally rare.

**Case presentation:**

A 13-year-old previously healthy female presented with a one-week history of progressive pallor, jaundice, and fatigue. Initial laboratory tests revealed severe anemia (Hb 5.6 g/dL), elevated LDH, indirect hyperbilirubinemia, undetectable haptoglobin, and a positive direct antiglobulin test (DAT) for IgG, confirming warm AIHA. Imaging studies, including pelvic ultrasound and MRI, identified a large complex cystic ovarian mass, consistent with a mature cystic teratoma. The patient underwent exploratory laparotomy with right salpingo-oophorectomy without requiring blood transfusions. Postoperatively, there was complete resolution of hemolysis, normalization of laboratory values, and no recurrence over a 2-month follow-up period.

**Conclusion:**

This case highlights a diagnostic challenge and underscores the importance of recognizing paraneoplastic AIHA in children, even in the context of benign tumors. It also supports a potential autoimmune–tumor association, emphasizing the value of timely diagnosis and surgical intervention.

## 1 Introduction

Autoimmune hemolytic anemia (AIHA) is considered rare but at the same time it is a serious condition in pediatric populations (1), has an estimated incidence equivalent to one case per 125,000–500,000 individuals (2). It is mediated by autoantibodies, which are misdirected immunoglobulins that bind to red blood cell antigens and lead to their premature destruction (3), and it is classified based on the thermal antibody activity into warm or cold types (4). AIHA can be primary (without an identified cause) or secondary due to infection, autoimmune condition, drugs or malignancies (3). Mature cystic teratomas are benign germ cell tumors that contain differentiated tissues like hair or bone, and in most cases, they discovered incidentally during imaging due to other medical causes (5). They are relatively common in adolescent females but are only infrequently implicated in systemic paraneoplastic phenomena (6). In contrast, the association between AIHA and solid tumors, especially benign neoplasms such as ovarian mature cystic teratomas in pediatric patients, is exceptionally rare, with only a few cases reported in the literature (7). There are several hypotheses to explain this association between teratomas and AIHA, such as antigenic cross-reactivity between tumor cells and red blood cells, direct production of autoantibodies by the tumor, or tumor-induced alterations in red blood cell surface antigens, all of which may contribute to the development of paraneoplastic AIHA (8, 9). Several case reports, starting in the mid-20th century, have documented the resolution of autoimmune hemolytic anemia after surgical excision of the ovarian dermoid cyst, supporting the theory of a tumor-induced autoimmune response (10–12). In this case, we present rare paraneoplastic manifestation of AIHA and emphasize the importance of considering occult neoplasms in the differential diagnosis of pediatric hemolytic anemia, particularly in patients with a family history of systemic lupus erythematosus (SLE).

## 2 Case report

A 13-year-old previously healthy female presented to the emergency department on April 24, 2025, with acute onset of progressive pallor and jaundice. Symptoms were progressive and began interfering with daily activity. Her symptoms began approximately one week prior and were associated with a noticeable reduction in energy levels. She denied abdominal pain, vomiting, diarrhea, or bleeding tendencies. There was no history of recurrent infections or other constitutional symptoms. She reported intermittent, mild joint pain over the past year, mainly in the knees. The pain was non-inflammatory in nature, not associated with swelling, redness, or morning stiffness, and did not interfere with daily activities. In addition, she described occasional night sweats that started one-month before her symptoms began, typically waking up to find her clothes slightly damp and feeling hot enough to remove the blanket, though not accompanied by fever or weight loss. She reported rare, brief peripheral visual phenomena occurring during sleep onset, consistent with benign hypnagogic illusions. These were not accompanied by disorientation, mood changes, cognitive disturbances, or neurologic findings, and were deemed non-pathological.

Menstrual history was unremarkable. She had menarche at age 12, with regular monthly cycles lasting five days, and no complaints of menorrhagia or dysmenorrhea. There was no personal history of autoimmune disease. However, her family history was notable for breast cancer on both maternal and paternal sides, and an aunt diagnosed with systemic lupus erythematosus (SLE) seven years ago.

On physical examination, the patient appeared pale but was well-hydrated and alert. Multiple ecchymotic patches were observed over the lower limbs. Abdominal examination revealed a soft, lax abdomen with no tenderness, masses, or organomegaly. No hepatosplenomegaly was noted. The musculoskeletal examination, including the pediatric gait, arms, legs, and spine (PGALS) screening, was normal. There was no peripheral lymphadenopathy. Cardiovascular and respiratory examinations were unremarkable, and neurological examination was grossly intact, with no focal deficits or signs of raised intracranial pressure.

Initial laboratory investigations revealed a hemoglobin level of 5.6 g/dL. Markers of hemolysis were elevated, including lactate dehydrogenase (700 U/L), uric acid (560 µmol/L), and undetectable haptoglobin. Total bilirubin was elevated at 102 µmol/L, with a predominantly indirect component (direct bilirubin: 9 µmol/L). The reticulocyte count was elevated to 6.1%, indicating appropriate bone marrow compensation. Peripheral blood smear demonstrated spherocytes, nucleated red blood cells (nRBCs), and auto-agglutination. A direct antiglobulin test (DAT) was positive for IgG, consistent with warm autoimmune hemolytic anemia (AIHA).

**Figure 1:**
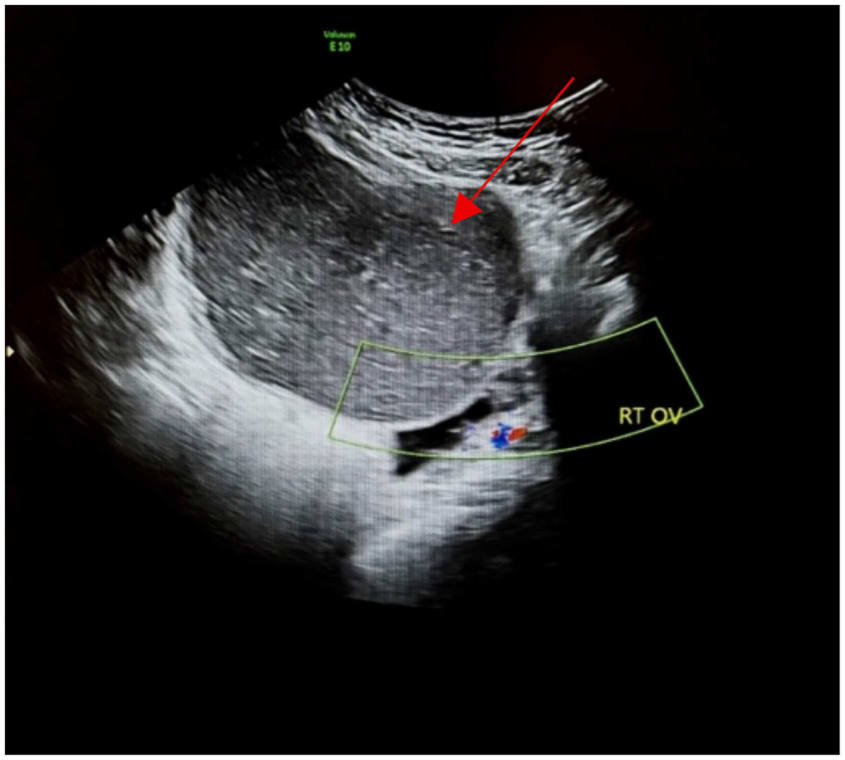
Transabdominal ultrasound of the right ovary (RT OV) demonstrates homogeneous hypoechoic cystic adnexal mass without internal increased vascularity (red arrow).

**Figure 2:**
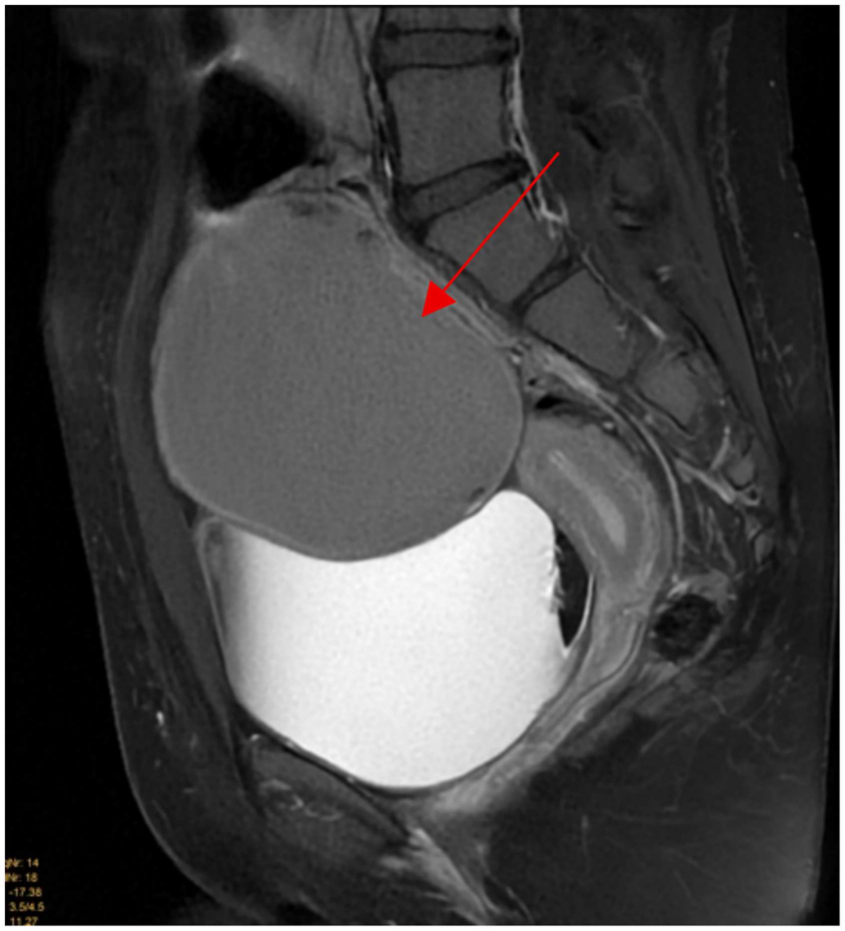
Sagittal T2 fat suppressed weighted images shows 9 × 8 cm well defined homogeneous intermediate T2 signal intensity right adnexal cystic mass (red arrow).

Pelvic ultrasound identified a large, well-defined, predominantly cystic mass arising from the right ovary, with internal septations and echogenic components suggestive of fat content (Figure 1). MRI of the pelvis confirmed a complex multilocular cystic lesion, measuring approximately 8–9 cm (Figure 2). No solid enhancing nodules or invasive features were seen. There was no evidence of pelvic lymphadenopathy or bone marrow infiltration.

Tumor markers, including β-human chorionic gonadotropin (β-HCG) and alpha-fetoprotein (AFP), were within normal limits. Autoimmune serology revealed a weakly positive antinuclear antibody (ANA), while anti-dsDNA and ENA panels were negative. Virologic studies for Epstein–Barr virus (EBV) and cytomegalovirus (CMV) were pending at the time of evaluation. Transthoracic echocardiography showed a small atrial septal defect (ASD II) with left-to-right shunting, preserved biventricular function, and no pericardial effusion.

**Figure 3:**
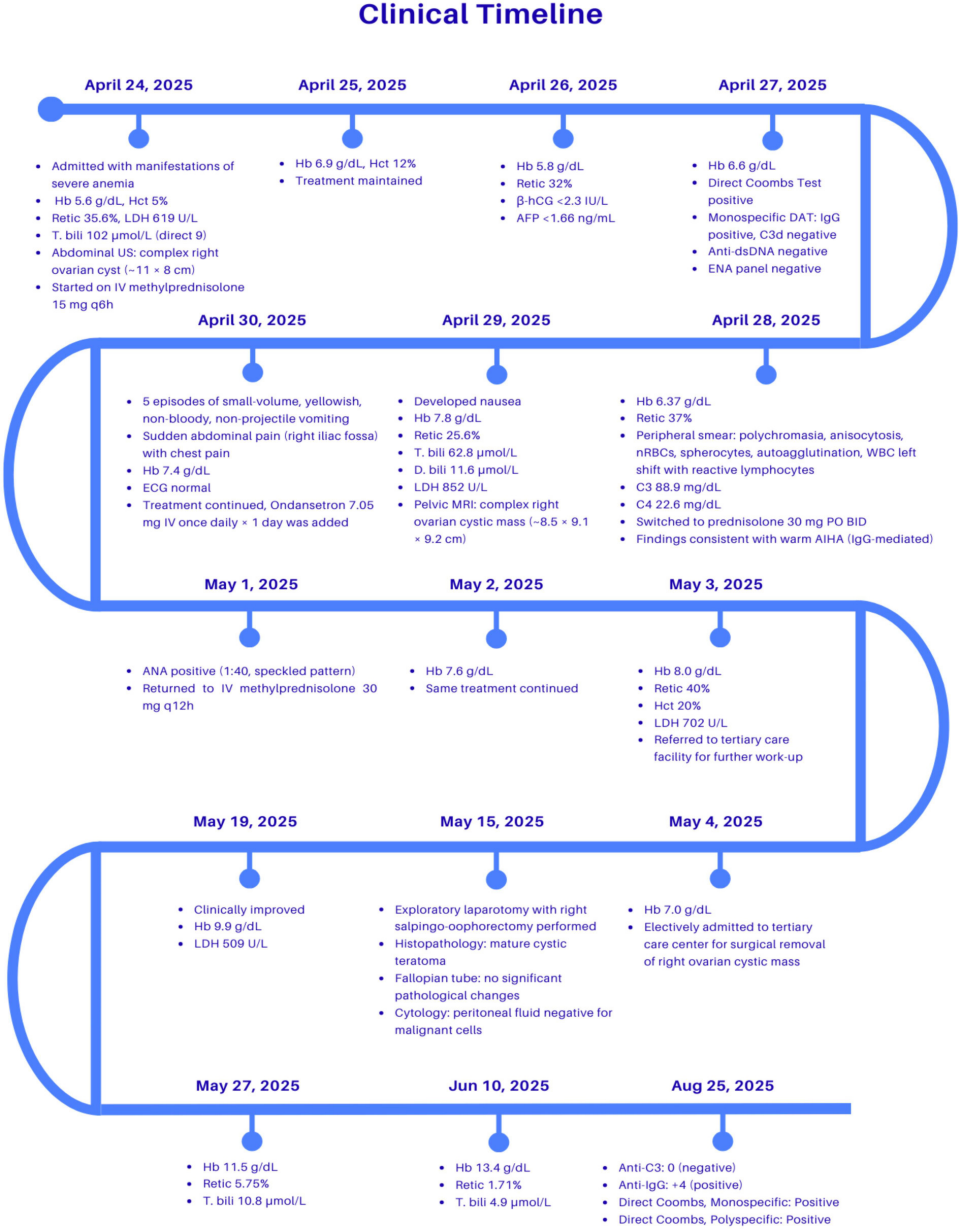
Clinical timeline demonstrating the chronological course of the patient's illness, showing key laboratory results, imaging findings, therapeutic interventions, and clinical outcomes from admission to recovery. Hb, Hemoglobin; Hct, Hematocrit; Retic, Reticulocyte count; LDH, lactate dehydrogenase; T. bili, Total bilirubin; D. bili, Direct bilirubin; US, Ultrasound; MRI, Magnetic resonance imaging; ECG, Electrocardiogram; ANA, Antinuclear antibody; DAT, Direct antiglobulin test (Coombs test); IgG, Immunoglobulin G; C3/C3d, Complement component 3; Anti-C3, Antibody against complement 3; Anti-IgG, Antibody against immunoglobulin G; Monospecific/Polyspecific Coombs, Variants of DAT detecting specific (IgG, C3) or combined antibodies; dsDNA, Double-stranded DNA; ENA, Extractable nuclear antigen; β-hCG, Beta-human chorionic gonadotropin; AFP, Alpha-fetoprotein; PO, Oral; IV, Intravenous; BID, Twice daily; q6/q12h, Every 6 or 12 h.

A detailed clinical course, including laboratory findings and management steps, is illustrated in a timeline (Figure 3).

## 3 Therapeutic intervention

Laboratory investigations confirmed the presence of severe anemia, with a hemoglobin level of 6.3 g/dl, and a diagnosis of warm autoimmune hemolytic anemia (AIHA) was established. The patient was admitted and initiated on high-dose intravenous methylprednisolone at a dose of 30 mg every 12 h, which was subsequently transitioned to oral prednisolone at a dose of 30 mg every 12 h. Over the course of five days, she exhibited a steady improvement in hemoglobin levels, reaching 7.8 g/dL, notably without the need for blood transfusions. Due to social circumstances, the family elected to transfer the patient to a tertiary care center for further management.

At the tertiary center, the patient underwent multidisciplinary evaluation, and the ovarian mass was determined to be a likely contributing factor to her persistent hemolysis. A surgical excision was planned, and she underwent an exploratory laparotomy with right salpingo-oophorectomy. Intraoperative findings were consistent with a mature cystic teratoma. This was confirmed by the surgical pathology report, which revealed a mature cystic teratoma, and the cytology report showed peritoneal fluid negative for malignant cells. Postoperatively, the patient demonstrated significant clinical and hematologic improvement. By the fourth postoperative day, her hemoglobin level had risen to 9.9 g/dL. Markers of hemolysis declined. No further immunosuppressive therapy or transfusion was required, and the patient remained well on outpatient follow-up with no recurrence of anemia.

## 4 Follow-up and outcomes

The patient underwent laparotomy with right salpingo-oophorectomy, and histopathological examination of the excised mass confirmed the diagnosis of a mature cystic teratoma. Following the surgery, there was complete resolution of hemolysis, with rapid clinical improvement and normalization of vital signs. Hemoglobin levels improved without the need for further blood transfusions, and follow-up laboratory testing showed normalization of lactate dehydrogenase (LDH), indirect bilirubin, and reticulocyte count, confirming laboratory improvement. At both 1-month and 2-month follow-up visits, the patient remained stable and asymptomatic, with no recurrence of hemolysis. Routine complete blood counts and markers of hemolysis remained within normal limits. Given her weakly positive ANA and a family history of autoimmune disease, long-term autoimmune screening was initiated and remains ongoing in the outpatient setting. The Direct Antiglobulin Test (DAT) remained positive, with a strongly positive anti-IgG (+4) and negative anti-C3, confirming persistent warm AIHA. This finding suggests that, although tumor removal may have addressed the potential paraneoplastic trigger, circulating autoantibodies were still present. The persistence of DAT positivity may reflect delayed antibody clearance or the possibility of co-existing primary AIHA.

## 5 Discussion

The association between ovarian teratoma and autoimmune hemolytic anemia (AIHA) was first described by West-Watson and Young in 1938 (13). Although ovarian teratomas are relatively frequent, their association with hemolytic anemia is uncommon. And such presentations are even more uncommon in pediatric patients, as documented in only a few reports. Goyal et al. (14) a 9-year-old boy developed AIHA that was refractory to corticosteroid tapering and only resolved after surgical excision of the sacrococcygeal teratoma yet the direct Coombs test remained positive in follow-up (14). Similarly, a case of a 3-year-old girl with AIHA secondary to a sacrococcygeal germ cell tumor was reported, where hemolysis resolved following multimodal therapy and surgical resection, further supporting the paraneoplastic mechanism in younger patients (15). Compared to previous pediatric reports describing sacrococcygeal teratoma–associated AIHA, our case represents one of the youngest reported adolescent females with ovarian mature cystic teratoma presenting with this rare association, further supporting molecular mimicry as the most likely underlying mechanism.

To our knowledge, this is one of the few documented pediatric cases where AIHA resolved following resection of a benign ovarian teratoma, supporting a paraneoplastic autoimmune mechanism in an adolescent population. In all cases, tumor removal was the definitive treatment, suggesting that early surgical intervention should be considered when AIHA is refractory to immunosuppressive therapy in the presence of a teratoma. The exact mechanism underlying the association between teratomas and autoimmune hemolytic anemia (AIHA) remains unclear. However, several hypotheses have been proposed in the literature. One possibility is tumor-induced autoimmunity, in which the teratoma triggers an immune response *that cross*-reacts with red blood cell antigens—a mechanism consistent with paraneoplastic syndromes (8, 9). This phenomenon is also described as molecular mimicry, where tumor-derived antigens share structural similarities with red blood cell antigens, leading to the production of autoantibodies that mistakenly target both.

Another potential factor is immunogenetic predisposition, particularly in patients with a family history of autoimmune diseases such as systemic lupus erythematosus (SLE). These theories highlight the complex interplay between neoplastic and autoimmune processes and may explain why tumor resection often leads to resolution of hemolysis in reported cases. While our patient only presented with autoimmune hemolytic anemia (AIHA), it is important to note that ovarian teratomas have been associated with a broader spectrum of paraneoplastic syndromes described in the literature (16) (Table 1). Neurological syndromes include anti–N-methyl-D-aspartate receptor (NMDAR) encephalitis, limbic encephalitis, encephalomyelitis, neuromyelitis optica spectrum disorder [NMOSD; associated with aquaporin-4 (AQP4) antibodies], myelin oligodendrocyte glycoprotein (MOG)-associated disease, glial fibrillary acidic protein (GFAP) astrocytopathy, progressive cerebellar degeneration, and opsoclonus–myoclonus syndrome (OMS) (17). Non-neurological manifestations, though rare, have also been described, including immune thrombocytopenia (ITP), aplastic anemia, hemophagocytic lymphohistiocytosis (HLH), polymyositis, dermatomyositis, seronegative polyarthritis, and tenosynovitis (16, 18–20). Endocrine and metabolic disturbances such as paraneoplastic hypercalcemia and syndrome of inappropriate antidiuretic hormone secretion (SIADH), as well as vascular events like paraneoplastic venous thrombosis, have also been reported (19, 21).

**Table 1:** Reported paraneoplastic syndromes associated with ovarian teratoma.

Category	Syndrome	Key Features
Neurological	Anti-NMDAR encephalitis, Limbic encephalitis, Encephalomyelitis, Cerebellar degeneration/Opsoclonus-myoclonus, NMOSD (AQP4), MOG-associated disease, GFAP astrocytopathy	Psychiatric changes, seizures, memory loss, abnormal movements, ataxia, optic neuritis, encephalopathy
Hematologic	Autoimmune hemolytic anemia (AIHA), Immune thrombocytopenia (ITP), Aplastic anemia, HLH	Hemolysis, cytopenias, jaundice, bleeding tendency, fever, splenomegaly
Rheumatologic/Musculoskeletal	Polymyositis, Dermatomyositis, Seronegative polyarthritis, Tenosynovitis	Proximal muscle weakness, ↑CPK, rash, arthritis, joint pain/swelling
Endocrine/Metabolic	Hypercalcemia, SIADH	Hypercalcemia symptoms (fatigue, confusion), hyponatremia due to inappropriate ADH
Vascular (rare)	Thrombosis (e.g., internal jugular vein)	Venous thrombosis as initial manifestation

The standard first-line therapy for AIHA is corticosteroids, which may induce partial or complete remission in primary cases. However, similar to other teratoma-associated reports, our patient showed only transient improvement with steroids, and hemolysis persisted until tumor removal. This underscores the fact that immunosuppressive therapy alone is insufficient in paraneoplastic AIHA, where definitive management requires surgical excision of the underlying tumor (22–24).

This case highlights several key clinical considerations. In the setting of unexplained hemolysis that proves refractory to standard immunosuppressive therapy, pelvic imaging should be considered to investigate potential underlying neoplasms, such as ovarian teratomas. Early identification of such masses may allow for timely surgical intervention and, in some cases, lead to complete hematologic remission. Managing prolonged hemolysis also requires careful planning to avoid unnecessary transfusions. This can be achieved through close monitoring of laboratory trends, individualized transfusion thresholds, and early involvement of a multidisciplinary team to guide further evaluation and intervention. Additionally, exploring a family history of autoimmune diseases, including systemic lupus erythematosus (SLE), may help identify an underlying immunogenetic predisposition. This approach is particularly relevant in young patients and may inform the diagnostic process in cases of atypical or treatment-resistant AIHA. Furthermore, in our patient, no splenomegaly was detected clinically or radiologically. This finding is consistent with secondary AIHA, as hepatosplenomegaly is more commonly reported in primary AIHA cases. The absence of splenic enlargement further supports the paraneoplastic nature of the hemolysis in this case (25). In our patient, hemolysis did not recur following tumor resection; however, the direct Coombs test remained weakly positive, a finding that has also been reported in previous cases (14, 15).

## 6 Conclusion

This case highlights that autoimmune hemolytic anemia (AIHA) can occur as a paraneoplastic phenomenon, even in association with benign tumors such as mature cystic teratomas. Early clinical recognition, prompt imaging, and definitive surgical excision of the underlying lesion may lead to complete resolution of hemolysis and obviate the need for prolonged immunosuppressive therapy. In pediatric patients presenting with unexplained or treatment-resistant AIHA, especially adolescent females, clinicians should maintain a high index of suspicion for underlying neoplasms. Even benign tumors may trigger immune-mediated hemolysis, and timely surgical removal can result in hematologic remission. Moreover, the presence of a family history of autoimmune diseases and malignancies may suggest an underlying genetic or immunologic predisposition, underscoring the importance of long-term autoimmune surveillance in such patients.

## Patient perspective

It was the most difficult period I've ever gone through. As a mother, watching my daughter become pale, tired, and not knowing why was terrifying. The worst part was the uncertainty even the doctors were unsure at first, and that scared me even more. Every day I felt like walking in the dark, hoping for answers. When we finally got a diagnosis, I cried not out of fear, but relief. Finally, we knew what was wrong, and that *something could be done. After the surgery, she started to come back to life. I will always be grateful for the care she received and for having my daughter smile again.*

## Data Availability

The raw data supporting the conclusions of this article will be made available by the authors, without undue reservation.
