# Supplementary figures and images for: Case Report: Autoimmune hemolytic anemia associated with ovarian teratoma in a 13-year-old: a rare paraneoplastic presentation
Source: Front Pediatr. 2025 Oct 27;13:1700443. doi: 10.3389/fped.2025.1700443 (PMC12597929; doi:10.3389/fped.2025.1700443)

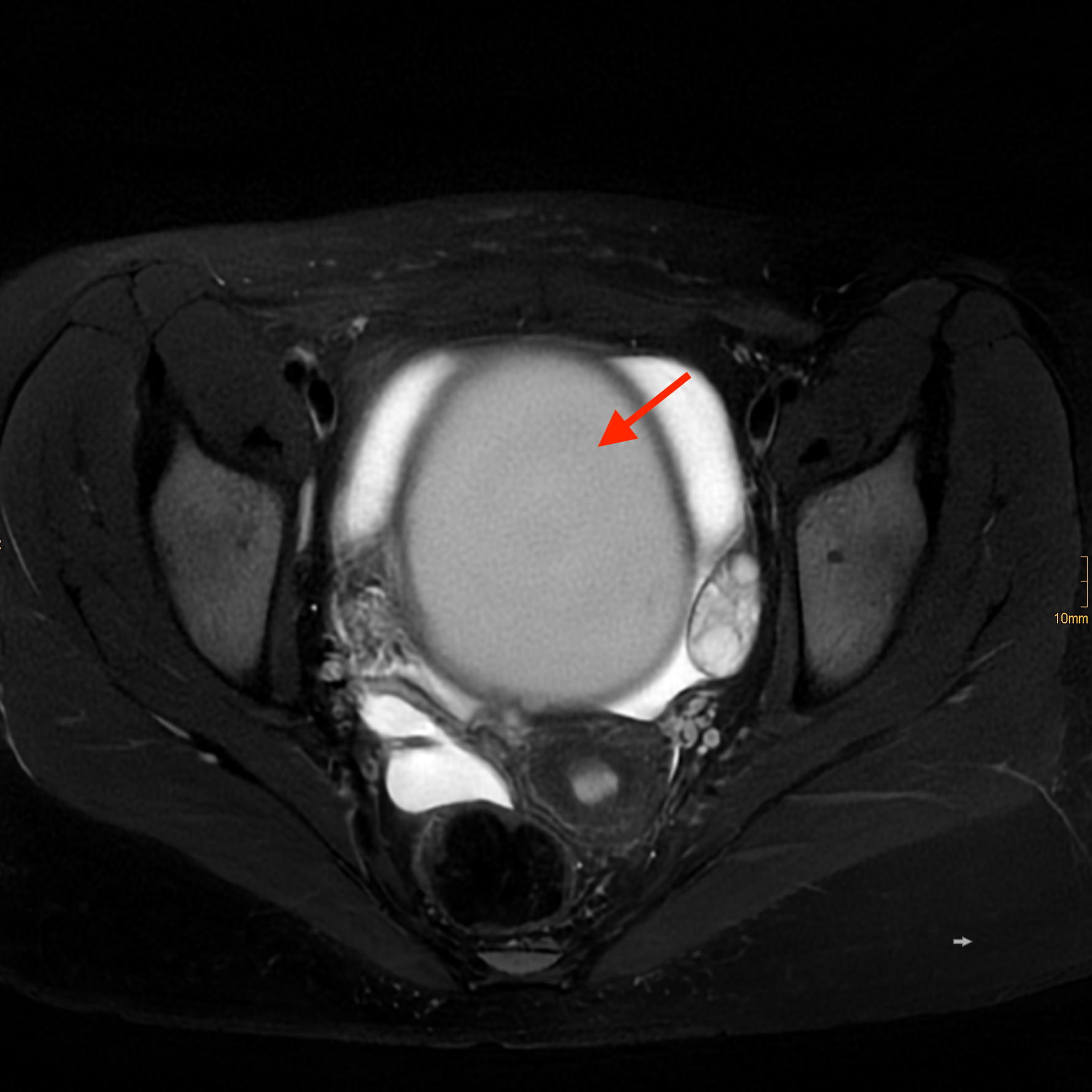

Supplement: Supplementary file 1 [file Image1.tiff]

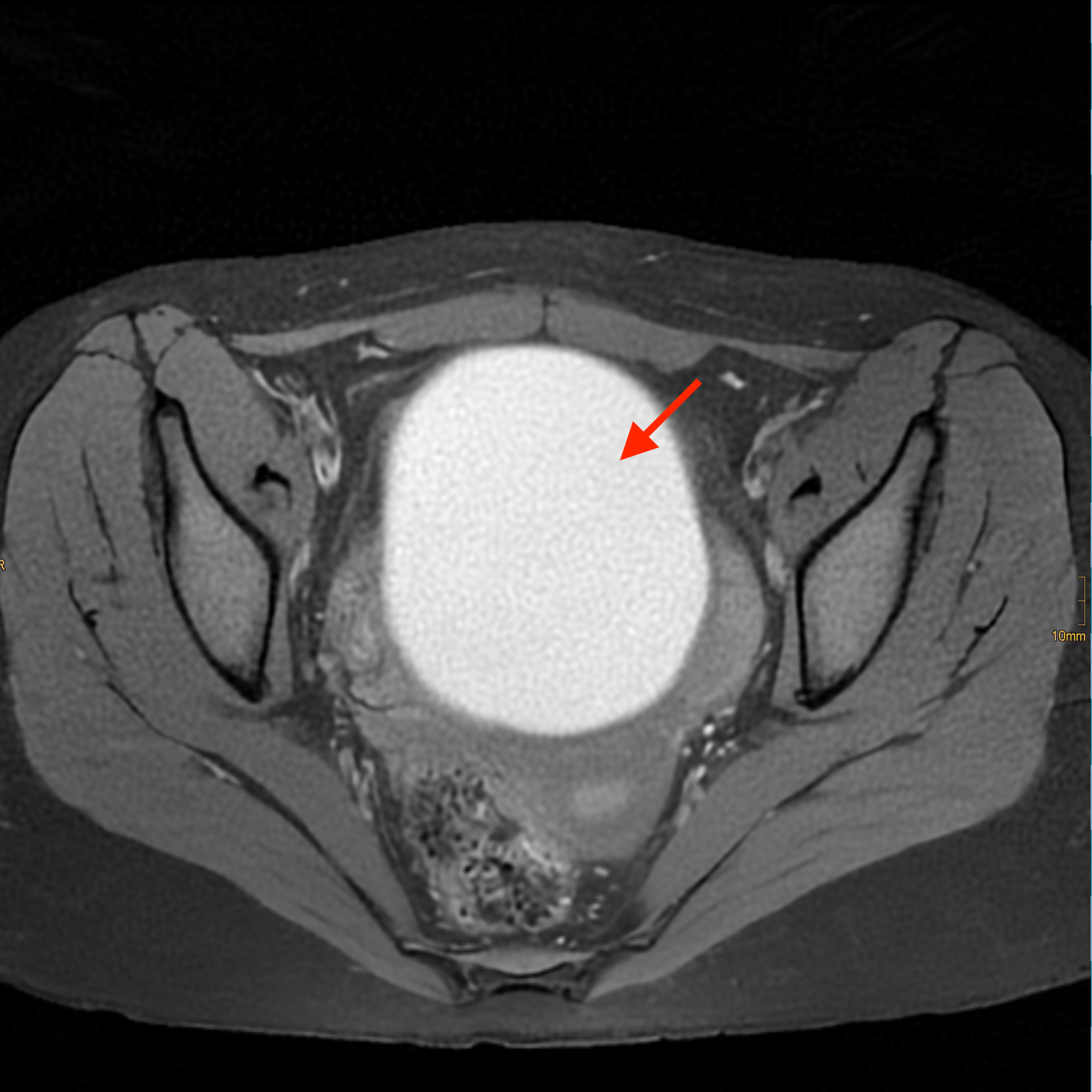

Supplement: Supplementary file 2 [file Image2.tiff]

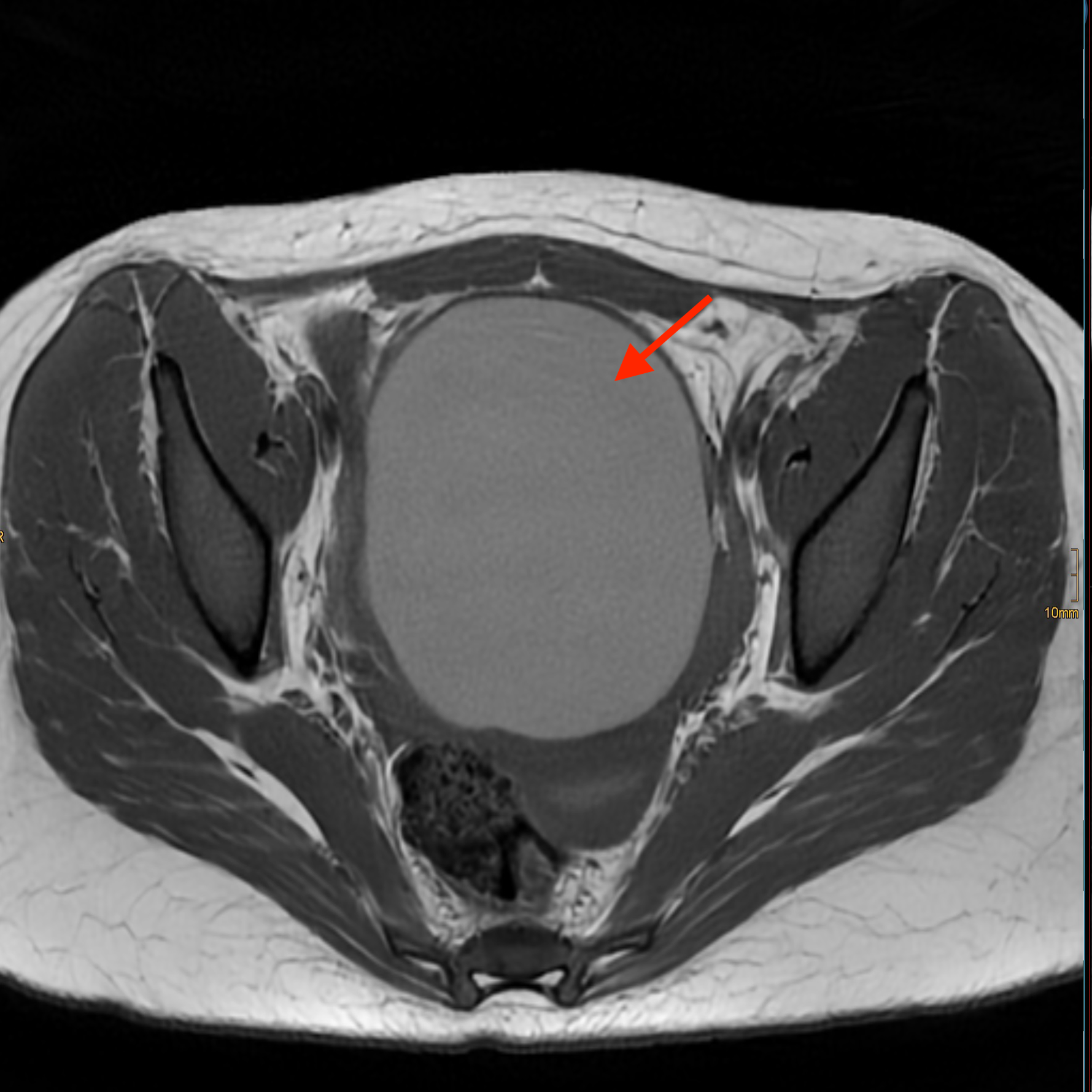

Supplement: Supplementary file 3 [file Image3.tiff]

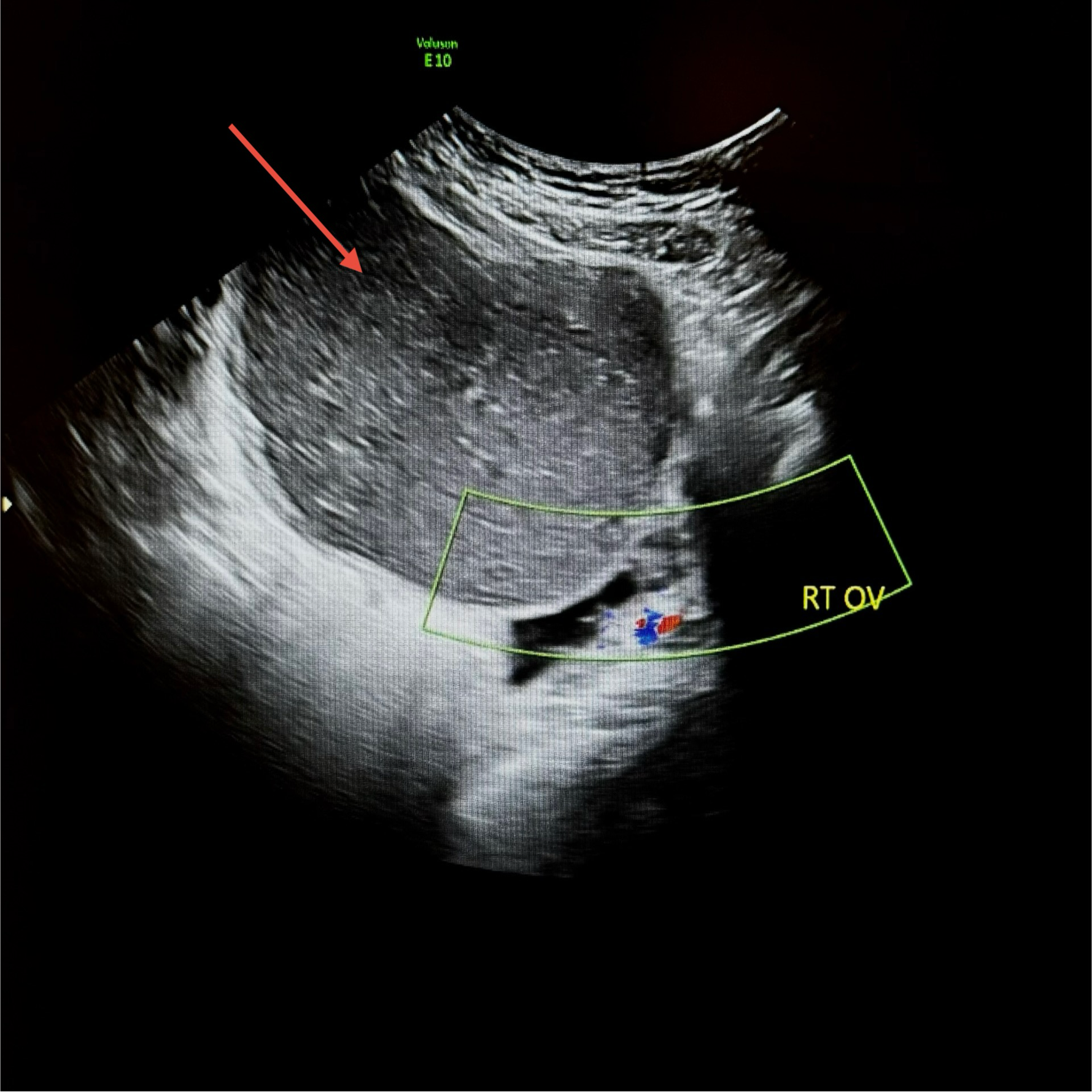

Supplement: Supplementary file 4 [file Image4.tiff]
